# Platelet FcɣRIIa Expression Refines Clinical Risk Assessment After Myocardial Infarction

**DOI:** 10.1002/ccd.70691

**Published:** 2026-06-16

**Authors:** David J. Schneider, Sean R. McMahon, Dominick J. Angiolillo, Alexander C. Fanaroff, Homam Ibrahim, Patrick K. Hohl, Brett L. Wanamaker, Mark B. Effron, Peter M. DiBattiste, behalf of the investigators

**Affiliations:** ^1^ Department of Medicine, Cardiovascular Research Institute The University of Vermont Burlington Vermont USA; ^2^ Department of Medicine Hartford Hospital Hartford Connecticut USA; ^3^ Department of Medicine, Division of Cardiology University of Florida Jacksonville Florida USA; ^4^ Department of Medicine University of Pennsylvania Philadelphia Pennsylvania USA; ^5^ Adventist Healthcare White Oak Silver Spring Maryland USA; ^6^ Division of Cardiovascular Medicine Maine Medical Center Portland Maine USA; ^7^ Division of Cardiovascular Medicine University of Michigan Ann Arbor Michigan USA; ^8^ John Ochsner Heart and Vascular Institute University of Queensland Ochsner Clinical School New Orleans Louisiana USA; ^9^ Prolocor Inc Philadelphia Pennsylvania USA

**Keywords:** biomarker, cardiovascular, platelet, prognosis

## Abstract

**Background:**

In patients with myocardial infarction (MI), quantifying expression of platelet FcɣRIIa (pFCG) stratifies risk of subsequent MI, stroke, and death.

**Aims:**

Assess the prognostic implications of clinical risk alone and in combination with the pFCG test.

**Methods:**

Patients (*n* = 749) were enrolled in a prospective non‐interventional trial during hospitalization with type 1 MI (ST elevation and non‐ST elevation). Inclusion criteria included ≥ 2 of the following clinical risk factors: age ≥ 65, multi‐vessel coronary artery disease, prior MI, chronic kidney disease, or diabetes mellitus. High and low pFCG (quantified by flow cytometry at a core laboratory) were defined by a prespecified threshold. The primary endpoint was the composite of MI, stroke, and death.

**Results:**

Distribution of clinical risk factors was 346 patients (45%) with 2 factors, 272 patients (36%) with 3 factors, and 131 patients (17%) with 4 or 5 factors. A greater number of risk factors was associated with a greater risk of the composite of MI, stroke, and death after 1 year. The event rate with 2 risk factors was 10.2%, with 3 factors it was 14.4%, and with 4/5 factors it was 35.6%. Across all cohorts of clinical risk, the pFCG test identified a higher‐risk and a lower‐risk cohort.

**Conclusions:**

Both clinical risk and the pFCG test assess prognosis. Results of the pFCG test refine the prognosis defined by clinical risk. The prognostic implications suggest that the pFCG test may be a useful tool to balance ischemic risk with that of bleeding to define treatment strategy.

**Clinical Trial Registration:**

https://clinicaltrials.gov/study/NCT05175261.

**Abbreviations**
DAPT: dual antiplatelet therapy
DM: diabetes mellitus
eGFR: estimated glomerular filtration rate
GRACE: Global Registry of Acute Coronary Events
MI: myocardial infarction
pFCG: platelet FcɣRIIa
SWEDEHEART: Swedish Web‐system for Enhancement and Development of Evidence‐based care in Heart disease Evaluated According to Recommended Therapies
TIMI: thrombolysis in myocardial infarction

1

While dual antiplatelet therapy (DAPT) with aspirin plus a P2Y_12_ inhibitor prevents thrombotic/ischemic events [1, 2, 3], DAPT is associated with a greater incidence of bleeding. There is substantial overlap between clinical characteristics associated with an increased risk of bleeding and ischemia, and more than one of these characteristics are often present in the same patient [4]. Because both bleeding and ischemic events (myocardial infarction [MI]) increase the risk of death [5], a risk‐informed, individualized approach to treatment has the potential to reduce both bleeding and ischemic events and thereby death [6, 7].

Clinical risk scores have been developed to assess the risk of subsequent ischemic events [4, 8, 9]. Three risk scores used in clinical practice are the TIMI (Thrombolysis in Myocardial Infarction) score [8], the GRACE (Global Registry of Acute Coronary Events) score [9], and the DAPT score [4]. Not surprisingly, these scores utilize common clinical characteristics associated with a greater risk of subsequent ischemic events. The Swedish Web‐system for Enhancement and Development of Evidence‐based care in Heart disease Evaluated According to Recommended Therapies (SWEDEHEART) registry associated cardiac risk factors with ischemic events in 103,934 individuals admitted for MI and managed invasively with coronary angiography from 2006 to 2014 [10]. They found that age ≥ 65 years, multivessel coronary disease (two or more vessels or left main with a stenosis ≥ 50% at coronary angiography), chronic kidney disease (estimated glomerular filtration rate [eGFR] below 60 mL/min/1.73 m^2^), diabetes mellitus (DM), and prior MI had the strongest association with the risk of ischemic events. Further, they found that ~54% had ≥ 2 risk factors and ~18% had no risk factors. Patients with a combination of risk factors had a greater risk of ischemic events.

Platelet FcɣRIIa (pFCG) is a biomarker that we have found to discriminate risk of subsequent ischemic events in two prospective non‐interventional studies [11, 12]. Multivariate analysis demonstrated that the pFCG test independently predicted risk of ischemic events [11, 12]. In this analysis, we assessed the risk of ischemic events (MI, stroke, and death) over 1 year based on clinical characteristics alone and in combination with the pFCG test in our 25‐center, 800‐patient study.

## 2 Methods

### 2.1 Participants

The study design has been previously reported [12]. Patients hospitalized for type 1 MI (ST elevation or non‐ST elevation) provided informed consent, and the research protocol was approved by either the site institutional review board or a central review board (WCG). To ensure a sufficient number of primary endpoints were accrued, results from the SWEDEHEART registry were used to identify and enroll patients at greater risk. Inclusion criteria required at least two of the following clinical risk factors: age ≥ 65 years, multi‐vessel coronary artery disease, prior MI, chronic kidney disease (defined as eGFR ˂60 mL/min/1.73 m^2^), or DM.

### 2.2 Quantification of pFCG

Citrate anticoagulated blood was taken at each clinical site within 2 weeks of enrollment, platelets were fixed with formaldehyde within 24 h after phlebotomy, and samples were shipped to a core laboratory and processed within 5 days of fixation. Analytic studies demonstrated that pFCG expression was stable for that interval [13]. The pFCG test quantifies FcɣRIIa on the surface of platelets with the use of flow cytometry. Flow cytometry output (mean fluorescence intensity) was converted to molecules of pFCG/platelet with the use of standardized beads (Quantibrite, BD Biosciences). A prespecified threshold (1750 molecules/platelet) was used to identify high and low pFCG. The threshold used to define high and low pFCG was prespecified for the two prospective studies (11,12) based on preliminary evaluation performed on a limited number of patients who had experienced multiple MIs in 1 year compared with patients who had experienced only 1 MI in a year. A midpoint value between the average expression in the two groups was selected as the threshold.

### 2.3 Outcomes

The primary endpoint was a composite of MI, stroke, and all‐cause death. Telephone follow‐up was performed every 6 months and used a standardized questionnaire. Patient‐reported events were confirmed by medical record review. Investigators identified clinical events (MI, stroke) by objective findings that included biomarkers (MI) or imaging (stroke).

### 2.4 Statistics

The trial design was event‐driven. Enrolled patients (projected to be 800 subjects) were planned to be followed until at least 80 ischemic events had occurred and the last subject has completed 18 months of follow‐up. This analysis focused on events during the first year of follow‐up.

A chi‐squared test was used to compare event rates between patients with low and high pFCG Trends across categories of risk (2 factors, 3 factors, and 4/5 factors). The Cochran−Armitage test for binary outcomes (e.g., event rates) and the Jonckheere−Terpstra test were used for the continuous outcome of age. Significance was defined as *p* < 0.05.

## 3 Results

As noted, the inclusion criteria required that participants had at least two of the defined clinical risk factors. The distribution of clinical risk factors among 749 patients was as follows: 346 (45%) had 2 risk factors, 272 (36%) had 3 risk factors, and 131 (17%) had 4 or 5 risk factors. Results from individuals with 4 or 5 risk factors were combined to provide a sufficient sample size for analysis. For the entire cohort, 217 patients (28%) had high pFCG. Among those with 2 risk factors, 25% (86 out of 346) had high pFCG. Among those with 3 risk factors, 29% (79 out of 272) had high pFCG. Among those with 4 or 5 risk factors, 37% (48 out of 131) had high pFCG (*p* < 0.001 for trend of percentage with high pFCG).

The clinical characteristics of the patients segregated into the 3 risk groups are shown in Table 1. STEMI was less common in those with more risk factors, and correspondingly, NSTEMI was more common. Notably, peripheral arterial disease (PAD), prior stroke, and insulin use were more common in patients with a greater risk. Among patients with a greater risk, treatment with clopidogrel was more common, and the use of prasugrel was less common.

**Table 1:** Clinical characteristics.

Characteristic	2 risk factors (*n* = 346)	3 risk factors (*n* = 272)	4/5 risk factors (*n* = 131)	*p*
Age (mean ± SD)	66 ± 10	70 ± 10	74 ± 7	
Gender (% male)	69% (238)	67% (181)	66% (87)	< 0.001
MI type				
STEMI	37% (128)	25% (67)	15% (20)	< 0.001
NSTEMI	67% (218)	75% (205)	85% (111)	< 0.001
HTN	78% (270)	93% (253)	97% (127)	< 0.001
DM	37% (128)	70% (189)	89% (116)	< 0.001
Insulin treatment	12% (40)	32% (86)	52% (68)	< 0.001
Active smoker	27% (93)	18% (48)	13% (17)	< 0.001
Hyperlipidemia	66% (227)	78% (211)	88% (115)	< 0.001
Prior MI	15% (53)	32% (87)	56% (73)	< 0.001
Prior CABG	6% (21)	13% (34)	37% (48)	< 0.001
Prior PCI	23% (81)	44% (119)	53% (70)	< 0.001
PAD	8% (27)	13% (34)	13% (17)	< 0.001
Prior stroke	6% (20)	11% (30)	18% (24)	< 0.001
Chronic kidney disease				
GFR < 60	10% (34)	35% (96)	82% (107)	< 0.001
ESRD	2% (8)	6% (17)	3% (4)	0.27
Medications				
Aspirin	93% (322)	92% (249)	92% (121)	0.70
Clopidogrel	51% (178)	59% (160)	66% (86)	0.003
Ticagrelor	25% (88)	23% (63)	20% (26)	0.20
Prasugrel	10% (34)	5% (13)	4% (5)	0.007
Anticoagulant	14% (47)	18% (49)	13% (17)	0.73
β‐blocker	88% (305)	89% (240)	86% (113)	0.67
CCB	18% (61)	26% (71)	21% (28)	0.12
Nitrates	28% (98)	32% (88)	47% (62)	< 0.001
ACEI/ARB	56% (194)	54% (147)	51% (67)	0.01
Diuretic	35% (120)	38% (102)	54% (71)	< 0.001
Lipid lowering	94% (324)	93% (253)	95% (124)	0.76

Abbreviations: ACEI = angiotensin converting enzyme inhibitor, ARB = angiotensin receptor blocker, CABG = coronary artery bypass surgery, CCB = calcium channel blocker, DM = diabetes mellitus, ESRD = end‐stage renal disease, GFR = glomerular filtration rate, HTN = hypertension, MI = myocardial infarction, NSTEMI = non‐ST elevation MI, PAD = peripheral arterial disease, PCI = percutaneous coronary intervention, STEMI = ST elevation MI.

**Figure 1:**
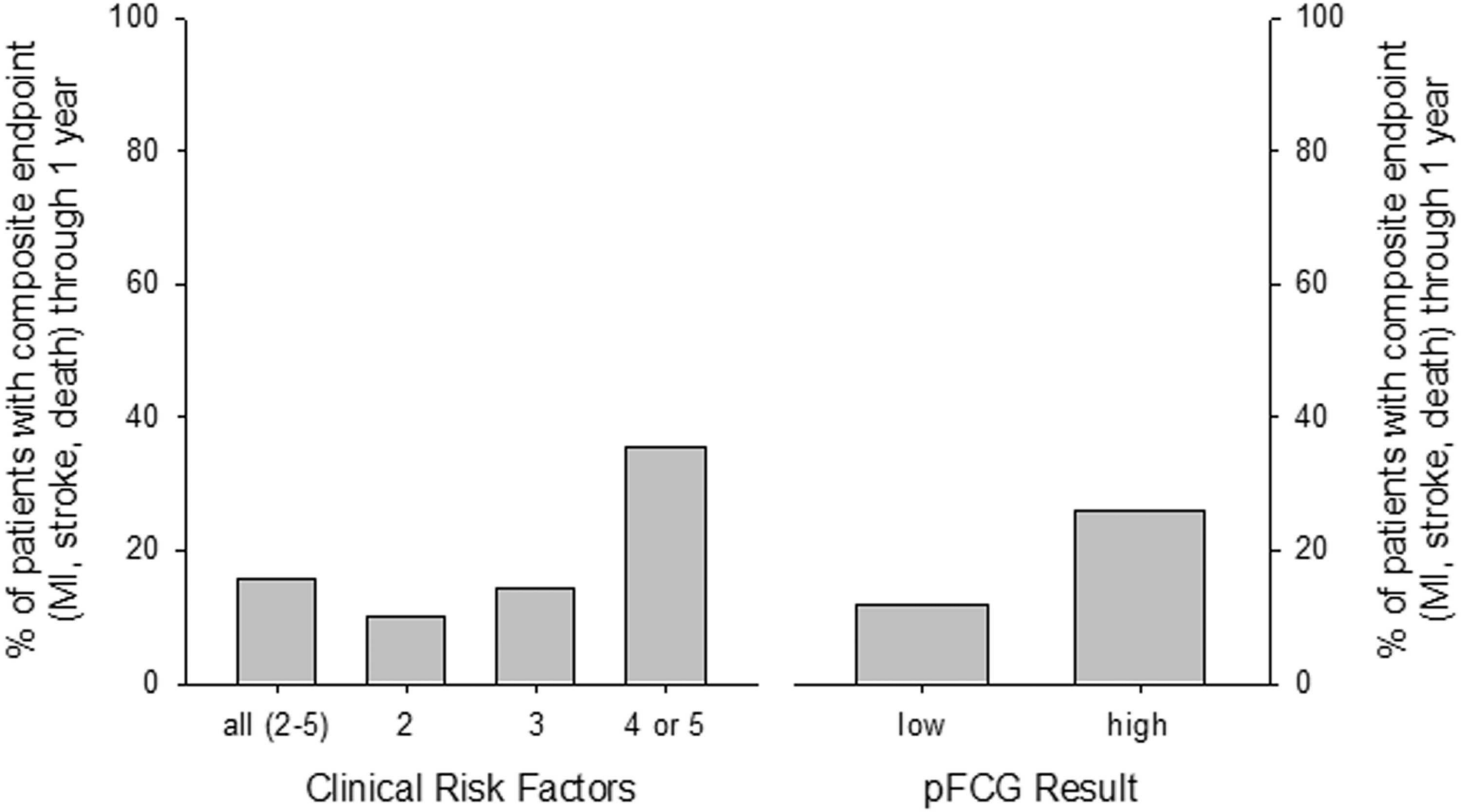
The 1 year incidence of the primary composite endpoint of MI, stroke, and death. The graph on the left shows results in patients divided by the number of clinical risk factors. The graph on the right shows results in patients divided into high and low pFCG test results. Patients with more clinical risk factors were at greater risk of the composite endpoint (*p* < 0.001 for trend). Patients with high pFCG had a greater risk of the composite endpoint (*p* = 0.001).

Both clinical risk factors and the results of the pFCG test discriminated risk (Figure 1). Notably, the presence of 4 or 5 clinical risk factors identified a patient group with extreme risk (1 year risk of the composite of MI, stroke, and death = 35.6%), whereas the 1‐year risk with 2 (10.2%) or 3 risk factors (14.4%) was substantially lower (*p* < 0.001 for trend).

**Figure 2:**
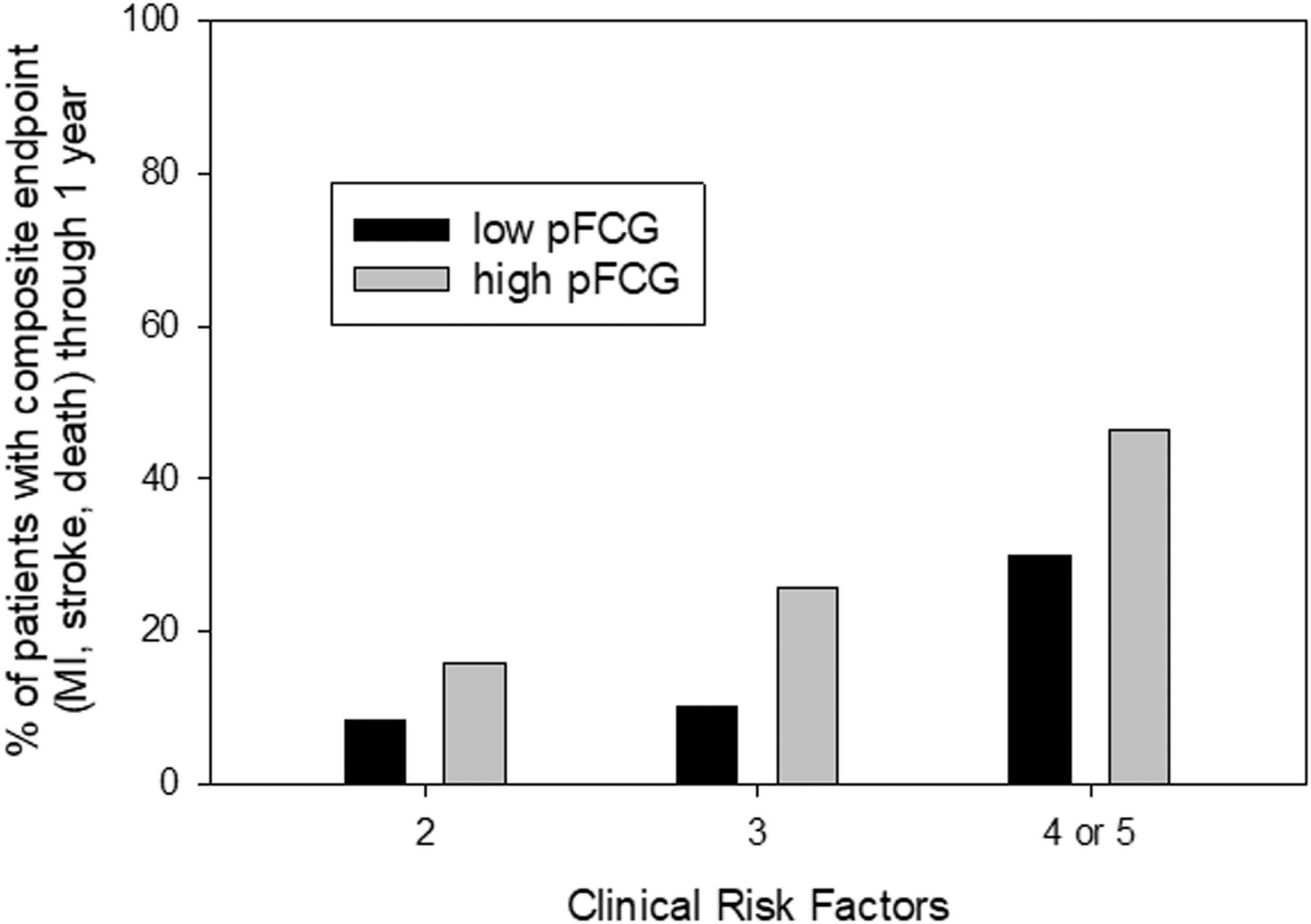
The 1 year incidence of the primary composite endpoint of MI, stroke, and death. Patients (*n* = 749) were divided into three groups based on the presence of clinical risk factors. The pFCG test was used to refine risk in each group. The absolute risk reduction (ARD) was 7.5% (*p* = 0.12) in patients with 2 clinical risk factors (*n* = 346), 15.5% (*p* = 0.019) for those with 3 risk factors (*n* = 272), and 16.5% (*p* = 0.37) for those with 4/5 risk factors (*n* = 131).

Patients with high pFCG had a greater risk than those with low pFCG in all subjects as well as in each of the clinical risk subsets (Figures 1 and 2, Table 2). For the primary composite endpoint, the absolute risk difference (ARD) between high and low pFCG was 14.1% for all subjects (*p* = 0.001). High pFCG had a similar association with clinical events regardless of clinical risk, and so the ARD comparing high with low pFCG increased with increasing clinical risk. Among patients with 2 clinical risk factors, the ARD was 7.5% (*p* = 0.12), for 3 risk factors, the ARD was 15.5% (*p* = 0.019), and for 4/5 risk factors, the ARD was 16.5% (*p* = 0.37). The relative risk of the primary endpoint in patients with high compared with low pFCG ranged from 1.9 in patients with 2 risk factors, to 2.5 with 3 risk factors, and was 1.6 in those with 4/5 risk factors.

**Table 2:** All treatment strategies: Event rate for death + MI through 1 year.

Clinical risk factors	*n*	All pFCG	Low pFCG	High pFCG	ARD	*p* value
All (2−5)	749	14.8	10.3	21.1	10.9	< 0.001
2	346	8.1	6.5	12.8	6.3	0.09
3	272	12.5	8.8	21.5	12.7	0.013
4 or 5	131	29	25.3	35.4	10.1	0.37

## 4 Discussion

Consistent with previous results [4, 8, 9, 10], we found that patients with a greater number of established clinical risk factors experienced an increased risk of the primary composite endpoint of MI, stroke, and death. Notably, patients with 4 or 5 risk factors in our enriched population exhibited extreme risk, with ~36% experiencing MI, stroke, or death over the course of 1 year. The pFCG test is an independent predictor of risk [11, 12]. Consistent with this analysis, the pFCG test identified patients with higher and lower risk in the entire study cohort as well as in each of the clinical risk categories. These results demonstrate the additive value of the pFCG test and its ability to refine risk assessment.

We adapted a traditional measure of clinical benefit, the number needed to treat, to assess the potential value of the pFCG test. The use of ticagrelor or prasugrel plus aspirin compared with clopidogrel combined with aspirin has been shown to reduce the incidence of MI, stroke, and death by 20% [2, 3]. Thus, treatment of patients with a high pFCG test result with ticagrelor or prasugrel plus aspirin rather than clopidogrel plus aspirin would be expected to reduce the 1‐year incidence of MI, stroke, and death by 20%. In our study, patients with high pFCG had an event rate of ~26%, and so a 20% risk reduction would decrease absolute risk by ~5%. Thus, for patients with high pFCG treated with clopidogrel, the number needed to test to reduce one ischemic event is 20. When all patients are considered and presuming all patients with high pFCG were treated with clopidogrel, the 5% absolute risk reduction in 30% of patients with high pFCG combined with no impact on the 70% with low pFCG, the overall absolute risk reduction would be expected to be approximately 1.6%. One fewer patient treated initially with clopidogrel would be expected to experience an ischemic event for every 64 patients tested and who were treated with a ticagrelor/prasugrel based on the outcome of the pFCG test.

Shortened treatment with DAPT (de‐escalation) reduces the risk of bleeding by approximately 50% with an absolute risk reduction from 5% to 2.5% [14, 15]. Because clinicians seek to balance the risk of bleeding and ischemic events, early de‐escalation of DAPT (after 3 months) would be an option in patients with low pFCG because of their lower risk of ischemic events. Among the 70% of patients with low pFCG, the number needed to test to support de‐escalation would be 40 patients. When all patients are considered (de‐escalation is not supported in the 30% with high pFCG), the number needed to test and treat in accordance with the test result in order to prevent one bleeding event is 57. Combining the results for ischemic events and bleeding events for all patients and presuming that all high pFCG patients are treated with clopidogrel, one ischemic or bleeding event could be prevented by testing 30 patients.

To put the results above in context, we compared our results with those of ticagrelor when tested in acute coronary syndromes. The PLATO (Study of Platelet Inhibition and Patient Outcomes) trial enrolled patients with acute coronary syndrome who were treated with PCI, CABG, or medical therapy alone. This design parallels that of our study. The number needed to treat to prevent one MI, stroke, or death was 53 [15]. A trial that uses the pFCG test to guide treatment will be needed to validate the estimated number needed to test values.

The prognostic implications of clinical risk factors seen in our study were comparable to those seen in the SWEDEHEART registry, a population‐based registry of patients evaluated invasively [10]. In the SWEDEHEART registry, 48.7% of patients had 0 or 1 risk factors. Enrollment criteria excluded these patients from our study. By contrast, only 6.6% of patients had 4 or 5 risk factors in the SWEDEHEART registry, whereas this group comprised 17% of our subjects. The risk seen in our study was modestly higher than that observed in the SWEDEHEART registry. For example, for patients with 4 or 5 risk factors, the range of risk in the SWEDEHEART registry was 18%−26%. The risk observed in our study was ~36%. Important differences between our study and the SWEDEHEART registry include a substantially smaller sample size of our study and the lack of a requirement for invasive evaluation in our study. We have reported that patients treated with medications alone (no PCI or CABG) were at a substantially higher risk and treated less intensively [16]. The inclusion of these patients is likely a substantial contributor to the greater risk observed in our study.

The greater prevalence of additional high‐risk characteristics likely contributed to the increased risk of cardiovascular events in patients with greater clinical risk. Notably, those with a greater number of clinical risk factors had a higher prevalence of PAD, prior stroke, and treatment with insulin. Each of these risk factors has been independently associated with a greater risk of cardiovascular events [17, 18, 19, 20, 21].

Treatment with clopidogrel was more common, and the aggregate treatment with ticagrelor and prasugrel was less frequent in patients with greater clinical risk. Two factors that likely contributed to this observation are the less common presentation with STEMI and the overlap between clinical risk factors for bleeding and ischemia [4].

Clinicians must balance the risk of ischemic events with that of bleeding because bleeding and recurrent MI have a similar association with risk of death [5]. There is substantial overlap between clinical risk factors associated with ischemic events and bleeding events. The results reported support the potential value of the pFCG test to inform clinical decision‐making by providing a powerful tool capable of identifying patients at higher and lower risk of ischemic events after MI. The combination of the pFCG test with a bleeding risk assessment has the potential to improve care by individualizing treatment decisions. The pFCG test has been designed to inform clinical decision‐making, providing results within 1–2 days. Ordering this test shortly after admission should allow clinicians to use results to guide treatment strategies before hospital discharge.

Limitations of this study include: (1) A single determination of the pFCG test shortly after MI that did not allow assessment of changes in this marker over time. Changes in this marker may influence risk. Future studies are needed to assess the prevalence of changes and the clinical implications of changes. (2) The enrollment of patients with at least 2 clinical risk factors limits extrapolation of these results to patients with 0 or 1 risk factor. A primary objective of this study was to validate the threshold used to define high and low pFCG. To ensure a sufficient number of events, clinical risk criteria were used to increase the total number of ischemic events. While this study (12) limits extrapolation of results to low‐risk patients, our first study (11) did not select patients based on clinical risk criteria and demonstrated comparable risk stratification with the pFCG test. (3) Risk of ischemic events was not assessed with established risk scores. This limits the ability to directly compare predictive power of the pFCG test with established clinical risk scores. The results of this study support plans for an interventional trial designed to use the pFCG test to guide individualized treatment.

In summary, both clinical risk and the pFCG test are powerful prognostic markers of ischemic risk. The pFCG test stratifies patients at higher and lower risk of subsequent cardiovascular events across the categories of clinical risk. The prognostic information provided by the pFCG test may be useful to clinicians as they balance risk of ischemic events with that of bleeding to define a treatment strategy. Future studies will be necessary to define specific treatment strategies based on the results of the pFCG test in combination with clinical risk assessment of ischemia and bleeding.

## Behalf of the investigators

Sites and investigators included: University of Vermont Medical Center; D. J. Schneider; Hartford Hospital, S. R. McMahon; University of Pennsylvania, A. Fanaroff; New York University, Claudia Serrano‐Gomez; Maine Medical Center, P. K. Hohl; University of Florida, D. J. Angiolillo; Ascension St Vincent, K. M. Ball; University of Michigan, B. L. Wanamaker; John Ochsner Heart and Vascular Institute, M. Effron; Lankenau Medical Center, T. A. Shapiro; St. Lukes Health System, B. Nolan, Prairie Education and Research Cooperative, A. Hassan; RWJ Barnabas Health, M. Cohen; Ascension St. John, D. Rodriguez; Ascension Sacred Heart, R. Amin; Sinai Center for Thrombosis Research, P. A. Gurbel; University of California Los Angeles, W. J. French; Ascension St. Thomas, T. Paul; Ascension Seton, R. Shutt; Our Lady of Lourdes Memorial Hospital, J. Pudusseri; Ascension Via Christi Hospital, W. Shaheem; Ascension Macomb, B. Mojares.

## Ethics Statement

The research protocol was approved by either the site's institutional review board or a central review board (WCG) for each participating site.

## Consent

All patients provided written informed consent.

## Conflicts of Interest

David J. Schneider is named inventor on patents (US 10,502,737, US 11,747,335 B2) that propose the use of FcγRIIa for assaying platelet reactivity and treatment selection. David J. Schneider and Peter M. DiBattiste are co‐founders of Prolocor. Dominick J. Angiolillo declares that he has received consulting fees or honoraria from Anthos, Bayer, Boehringer Ingelheim, Bristol‐Myers Squibb, Chiesi, Faraday, Idorsia, Johnson & Johnson, Novartis, Novo Nordisk, PLx Pharma, Sanofi, SFJ Pharmaceuticals, Vectura, Werfen; his institution has received research grants from Abbott, Amgen, AstraZeneca, Bayer, Chiesi, CSL Behring, DalCor Pharmaceuticals, Daiichi‐Sankyo, Edwards, Eli Lilly, Faraday, Janssen, Hikari DX, Novartis, Prolocor, Vertex. Mark B. Effron discloses that he receives a pension from and has equity in Eli Lilly and Company. Brett L. Wanamaker has received consulting fees from Asahi Intecc USA. The other authors declare no conflicts of interest.

## Data Availability

Data is available by contacting the principal investigator, David J. Schneider.
